# Ebracteolata cpd B causes ferroptosis and inhibits progression of lung adenocarcinoma

**DOI:** 10.3389/fphar.2026.1706564

**Published:** 2026-04-29

**Authors:** Yanan Du, Haidi Han, Yanjing Dong, Xiaoxiao Yin, Jingyu Zhu, Chonggao Yin, Haixia Wang

**Affiliations:** 1 Affiliated Hospital of Shandong Second Medical University, Weifang, Shandong, China; 2 College of Nursing, Shandong Second Medical University, Weifang, Shandong, China; 3 College of Traditional Chinese Medicine, Shandong Second Medical University, Weifang, Shandong, China

**Keywords:** *E. ebracteolata*, Ebracteolata cpd B, ferroptosis, GPX4, lung adenocarcinoma, SLC7A11

## Abstract

**Objectives:**

The study aims at examining the effects and mechanisms of *E. ebracteolata* containing serum and Ebracteolata cpd B on lung adenocarcinoma cells.

**Methods:**

The medicated serum with *E. ebracteolata* was categorized into groups of low, medium, and high concentrations. The proliferative potential of A549 cells was evaluated through EdU assay and colony formation. To characterize the migratory and invasive capacities, scratch and Transwell tests were applied. Network analysis and molecular docking were employed to generate testable hypotheses by predicting potential targets of ECB for LUAD. The effect of ECB on the thermal stability of SRC protein was assessed using the cellular thermal shift assay. Evaluation of ECB’s Assessment of Drug-like Properties and Safety. The CCK-8 assay served for evaluating A549 and BEAS-2B cells viability. The influence of ECB on the process that A549 cells were proliferated, invaded and migrated was evaluated employing the above-mentioned experimental method. *In vivo*, a mouse lung cancer model was built to verify how ECB affected the A549 cells growth. The levels of ROS, MDA, GSH, and the content of Fe^2+^ in A549 cells were measured. Observation of mitochondrial morphology by TEM. Western blot analysis assisted in measuring the expression of the proteins.

**Results:**

Compared to the control group, the serum with *E. ebracteolata* dose-dependently reduced A549 cells’ proliferation, invasion, and migration. Network analysis identified 9 core targets from the potential targets. Molecular docking suggested that ECB could potentially interact with these core targets. ECB significantly increased the thermal stability of SRC. ECB demonstrates favorable oral bioavailability and a good safety profile. CCK-8 results indicated that ECB exerted no significant toxicity on BEAS-2B cells, whereas it inhibited the viability of A549 cells. At its IC_50_, ECB inhibited A549 cell proliferation, migration, and invasion, and induced ferroptosis. Notably, Fer-1 inhibited ECB-induced ferroptosis in A549 cells. These effects were verified by measuring ROS levels, GSH reduction, MDA elevation, and intracellular Fe^2+^ content, and by TEM. GPX4 and SLC7A11 protein expression was reduced. Concurrently, ECB inhibited tumor-bearing mice’ relative tumor growth.

**Conclusion:**

ECB suppresses A549 cells proliferation, invasion, and migration as well as promotes ferroptosis.

## 1 Introduction

According to global statistics, lung cancer is the primary reason for cancer mortality, representing approximately 18.0% of all deaths attributed to cancer (Bray et al., 2024). Among all malignant tumors in China, lung cancer exhibits the highest occurrence and mortality (Han et al., 2024; Zheng et al., 2024). Among these, lung adenocarcinoma (LUAD) occupies about half of all lung cancer cases (Travis et al., 2011), and it demonstrates an even higher incidence rate in China (Han et al., 2024). Despite surgical resection being the primary therapeutic option for early-stage patients, many intermediate to advanced-stage patients lose the window for surgical intervention due to disease progression and exhibit poor tolerance to radiotherapy and chemotherapy (He et al., 2024; Vallieres et al., 2012). Therefore, we need to develop better therapeutic approaches. Traditional Chinese medicine (TCM), a treasure of the Chinese nation, not only significantly alleviates cancer-related symptoms with minimal adverse effects but also demonstrates clinically validated efficacy in suppressing tumor growth, gaining recognition within the oncology research community (Chen et al., 2024; He et al., 2016).

Ferroptosis, a kind of programmed cell death, is triggered by lipid peroxidation that depends on iron (Stockwell, 2022; Zheng and Conrad, 2020). The fast growth of cancer cells necessitates increased iron and lipid metabolism, making them more likely to undergo ferroptosis compared to normal cells (Li and Li, 2020; Wang et al., 2023). Recent years have witnessed a growing body of research demonstrating the complicated interplay between ferroptosis and cancer pathogenesis and progression (Zhou et al., 2024; Chen et al., 2021).

**FIGURE 1:**
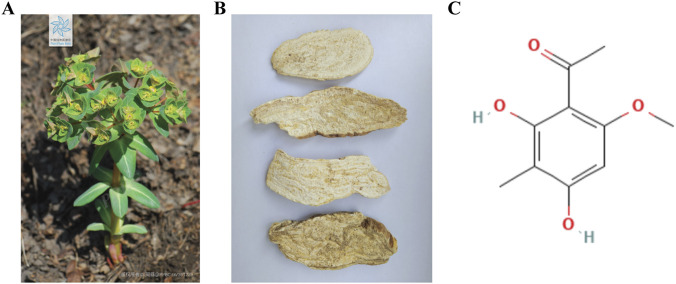
Morphological Characteristics of *E. ebracteolata*, Chemical Structure of Its Active Component ECB. Botanical characteristics of *E. ebracteolata*: **(A)** whole plant; **(B)** The dry root. **(C)** Chemical Structure of ECB.

*Euphorbia ebracteolata* Hayata (*E. ebracteolata*) is a traditional Chinese medicinal plant whose root is used for medicine (Figures 1A,B). Originally published in “Shennong Ben Cao Jing,” it is bitter, pungent, neutral, poisonous, and enters the lung, liver and spleen meridians. Its function is to expel water and drink, break the accumulation and kill worms (Commission, 2020). It is used for treating oedema and abdominal distension, phlegm and foodstuff accumulation, obstruction in the abdomen and tuberculosis. The Treatise on the Nature of Medicines: “Treating phlegm and drink, and obstruction in the abdomen.” According to relevant studies, the active components in *E. ebracteolata* show anti-tumor activity against HepG2 cells (Zhou et al., 2015) and breast cancer (BC) (Lv et al., 2019). However, the effect of *E. ebracteolata* on LUAD proliferation**,** invasion and migration has not yet been reported.

Ebracteolata cpd B (ECB), the main medicinal constituent in *E. ebracteolata* (Zhang et al., 2010), is an acetophenone compound (C_10_H_12_O_4_, Figure 1C). Relevant studies have elucidated the cytotoxic effect of ECB on HepG2 cells (Zhang et al., 2010), but have not well explored its inhibitory mechanism against LUAD.

This study seeks to test the effects of *E. ebracteolata* serum on A549 cells proliferation, invasion, and migration through a seropharmacological lens. Furthermore, network analysis and molecular docking in conjunction with *in vivo* and *in vitro* experiments are collectively employed for the elucidation of the regulatory mechanism of ECB against ferroptosis in LUAD. The anticipated outcomes are intended to establish a foundational research framework and theoretical basis for future scientific inquiries and clinical applications.

## 2 Materials and methods

### 2.1 Reagents and antibodies

EdU kit was bought from Beyotime (Shanghai, China). ECB (≥98% purity) was provided by Push (Chengdu, China). CCK-8 reagent was obtained from MeilunBio (Dalian, China). Reactive Oxygen Species (ROS) Assay Kit, Reduced Glutathione (GSH) Content Assay Kit, and Malondialdehyde (MDA) Content Assay Kit were provided by Boxbio (Beijing, China). The Cellular Iron Content Assay Kit was provided by Solarbio (Beijing, China). Ferroptosis inhibitor (Fer-1) and Erastin were purchased from MCE (Shanghai, China). The following primary antibodies were used: SLC7A11/xCT, GPX4 and SRC (both from Zenbio, Chengdu, China); GAPDH (Servicebio, Wuhan, China). Secondary antibodies were obtained from Huabio (Hangzhou, China).

### 2.2 Identification and preparation of drugs

*E. ebracteolata* was purchased from the TCM Pharmacy of Shandong Second Medical University Affiliated Hospital and authenticated by the Chief Physician of the Pharmacy. 32 g of the herb was soaked in 10 times its volume of distilled water for 30 min. It was first brought to a vigorous boil and then simmered on mild heat for 40 min. The decoction was filtered off. The herb residue was then decocted again in 8 times its volume of distilled water and filtered. The mixture of the two decoctions was concentrated to a final concentration of 0.032 g of raw herb per mL.

### 2.3 Grouping of rats and preparation of drug-containing serum

Female SD rats (SPF grade, n = 10), aged 6–7 weeks and weighing 200 ± 20 g, (Beijing Vital River Laboratory Animal Technology) were acclimatized in the laboratory animal center under standardized conditions of 23 °C ± 3 °C and 50% ± 10% relative humidity, with unrestricted access to food and water and freedom of movement. All animal experiments were conducted under Ethical Approval Number 2025SDL737.

Rats were randomized into two groups (n = 5): a blank serum group and a drug-containing serum group. Following a 1-week acclimatization period, the groups underwent twice-daily intragastric administration for seven consecutive days. The blank serum group was administered normal saline (10 mL/kg), comparatively, the drug-containing serum group was administered *E. ebracteolata* extract (0.32 g/kg) (Chen et al., 1994). Two hours after the final dose, the rats were administered pentobarbital sodium (2%, 40 mg/kg) by intraperitoneal injection for anesthetization. Blood samples collected via the abdominal aorta underwent 2 h of clotting at room temperature, followed by 10 min of centrifugation (3,000 × g, 4 °C). The supernatant serum was pooled for each group, heat-inactivated at 56 °C for half an hour, sterilized through 0.22 µm filtration, aliquoted, and maintained at −80 °C.

### 2.4 Grouping and preparation of drug-containing media

Cells were treated with serum diluted in RPMI-1640 medium as follows: Control group (Con: 10% blank serum), and Low- (LCG: 5%), Mid- (MCG: 10%), and High-concentration drug-containing serum groups (HCG: 15%) (Lin et al., 2023).

### 2.5 Cell culture

A549 and BEAS-2B cells lines (Wuhan Procell Life Science & Technology) were cultured in specific conditions: humidified, 37 °C and 5% CO_2_. A549 cells were grown in RPMI-1640 medium, whereas BEAS-2B cells were maintained in DMEM, with both media containing 10% FBS and 1% penicillin-streptomycin. Once 80%–90% confluence was achieved, the cells were passaged.

### 2.6 EdU cell proliferation assay

Cells on coverslips in 24-well plates were incubated with 1 mL of EdU working solution (1:1,000 dilution) per well for 2 h. After aspiration, cells were rinsed in PBS, and underwent 15 min of fixation in absolute methanol, and 40 min of permeabilization in 400 μL of 0.2% Triton X-100 per well in succession. Subsequent EdU staining followed the manufacturer’s protocol. An upright fluorescence microscope was employed for imaging EdU-positive cells.

### 2.7 Cloning experiment

Cells were plated at dishes (3 × 10^2^/dish) and cultured for 14 days, with the medium refreshed twice weekly. Subsequently, the cells received 20 min of fixation in methanol before Giemsa staining, followed by the quantification of the number of colonies.

### 2.8 Transwell invasion assay

Transwell inserts were prepared by coating them with 50 μL of Matrigel, followed by a 2 h incubation period. Cells, at a concentration of 4 × 10^4^ in 200 μL of medium, were introduced into the upper chambers, while the lower chambers were added with 500 μL of 1,640 medium supplemented with 10% FBS. Subsequent to 24 h of incubation, with non-invasive cells eliminated, the invaded cells were subjected to 15 min of fixation in methanol, Giemsa staining, and air-drying in succession. Images and quantification were performed on cells from five randomly selected fields per insert.

### 2.9 Scratch wound healing assay

In 6-well plates, cells were seeded and cultured until they achieved roughly 80% confluence. We used a 200 μL pipette tip to create a linear scratch in the center of each well, taking a straight edge for guidance. The wells underwent PBS rinses for debris elimination, and a medium containing the treatment was added. Initial images of the scratch were taken at 0 h, followed by incubation. After 24 h, migration was assessed by re-imaging scratch closure.

### 2.10 Drug target acquisition

The structure of ECB was obtained by utilizing the PubChem database (https://pubchem.ncbi.nlm.nih.gov/) and the SDF format was downloaded. The chemical formula was imported into SwissTargetPrediction database (http://swisstargetprediction.ch/), PharmMapper database (https://lilab-ecust.cn/pharmmapper/index.html/) for the prediction of putative drug targets.

### 2.11 Disease target acquisition

The GeneCards (https://www.genecards.org/) and OMIM (https://www.omim.org/) databases were used for the identification of targets potentially linked to LUAD. Retrieved targets were merged, deduplicated, and their official symbols standardized using UniProt. To manage the large GeneCards dataset, targets scoring above twice the median were retained for further analysis.

### 2.12 Construction of protein-protein interaction (PPI) network

The Venny2.1 online tool (https://bioinfogp.cnb.csic.es/tools/venny/) was employed to compare ECB targets and LUAD targets, aiming to identify potential overlapping genes.

### 2.13 PPI network analysis and core target acquisition

A PPI network constructed by importing the overlapping targets into the STRING database (https://cn.string-db.org/) was subsequently imported into Cytoscape 3.10.0 to receive topological analysis. Degree centrality (DC), closeness centrality (CC), and betweenness centrality (BC) were taken into account for the identification of key targets, which were computed with Cytoscape’s integrated tools and the CytoNCA plugin. Core targets were screened based on these metrics.

### 2.14 Gene ontology (GO) and kyoto encyclopedia of genes and genomes (KEGG) pathway enrichment analyses

GO and KEGG pathway enrichment analyses were carried out via the DAVID database (https://david.ncifcrf.gov/), followed by the visualization of the results as bubble charts via the Wei Sheng Xin (https://www.bioinformatics.com.cn/).

### 2.15 Molecular docking

The Protein Data Bank (PDB) database (http://www.rcsb.org/) provided the protein structures for the first nine key targets, while the molecular structure of ECB was entered into AutoDock Tools 1.5.7 for docking analysis. The results were displayed *via* PyMOL 2.4, while 2D interaction diagrams were generated with Discovery Studio 4.5. A structurally analogous compound of ECB was selected from the literature as a negative control and was subsequently docked into the binding site of the key target protein using identical parameters and protocols.

### 2.16 Cellular thermal shift assay (CETSA)

Cells were harvested and washed with PBS, followed by resuspension. After aliquoting, samples were heated in a water bath at 51, 54, 57, 60 °C and 63 °C for 3 min respectively, and then immediately cooled on ice. Following centrifugation, the supernatant was collected for protein extraction. The thermal stability of SRC was subsequently analyzed by Western blotting.

### 2.17 Assessment of drug-like properties and safety evaluation

Lipinski’s Rule of Five parameters (Molecular Weight (MW), Rotatable Bonds (Rbon), Hydrogen Bond Acceptors (Hacc), Hydrogen Bond Donors (Hdon), Lipophilicity (Log P)) of ECB were assessed using the SwissADME database (http://www.swissadme.ch/), so as to measure its drug-likeness and oral bioavailability potential.

The safety profile of ECB, including evaluation of hepatotoxicity, carcinogenicity, immunotoxicity, mutagenicity, cytotoxicity, and overall toxicity class, was predicted by virtue of the ProTox-II database (https://tox.charite.de/protox3/).

### 2.18 Detection of survival rate by CCK-8 method

Cells were placed in 96-well plates and left to incubate for 12 h. Subsequently, the culture medium in the ECB-treated group was replaced with complete medium that contained 10, 20, 40, 80, 160, and 320 μM ECB. Following another 24, 48, or 72 h of incubation, each well was added with CCK-8 reagent as per the producer’s protocol. Following a 1 h incubation, a microplate reader was adopted to detect the optimal density at 450 nm.

### 2.19 Measurement of ROS levels

A549 cells were plated in glass-bottom dishes to receive 24 h of culture. The mixture of cells with DCFH-DA working solution (1:1,000 diluted in serum-free medium) was incubated for 20 min. Subsequently, we adopted a confocal microscope for the observation and photographing of the cells.

### 2.20 Measurement of MDA, GSH, and Fe^2+^ levels

Supernatants from each experimental group were collected and aliquoted into both standard and sample wells as per the producer’s protocols for the respective assay kits. Absorbance values were determined at specified wavelengths (MDA: 450, 532, and 600 nm; GSH: 412 nm; Fe^2+^: 510 nm) utilizing a microplate reader. The samples’ concentrations of MDA, GSH, and Fe^2+^ were derived from the standard curves.

### 2.21 Observation of mitochondrial morphology by transmission electron microscopy (TEM)

After one night of fixation in 2.5% glutaraldehyde at 4 °C, A549 cells underwent 2 h of post-fixation with 1% osmium tetroxide, followed by ethanol dehydration and epoxy resin-embedding. Subsequent to sectioning, the ultrathin slices were stained with uranyl acetate and lead citrate, and measured via TEM for mitochondrial analysis.

### 2.22 Western blot analysis for intracellular protein expression levels

Total protein lysates were electrophoresed and moved onto PVDF membranes to receive indicated period of blockage in 5% skim milk. One night of incubation with primary antibody at 4 °C was followed by additional 2 h of incubation with secondary antibody at RT. Protein band was visualized with a gel imaging system, with gray values quantified with ImageJ software.

### 2.23 Establishment of mouse xenograft model

Female nude mice (SPF grade, 4 weeks old, 20 ± 2 g, n = 10) were acclimated for 3 days. Subcutaneous tumor models were established by injecting 0.1 mL A549 cells suspension (5 × 10^6^ cells/mL) into the scapular region of each mouse. Animal source and housing followed prior protocols.

### 2.24 Experimental grouping and drug administration

When tumor volume reached 50 mm^3^, mice were randomized into two groups (n = 5) using a block randomization method to ensure equal group sizes. The ECB treatment group received 10 mg/kg ECB by intraperitoneal injections every day for 14 days, and the control group administered the same volume of saline.

### 2.25 Monitoring of tumor volume changes and determination of tumor inhibition rate

Every other day, we measured and documented the long diameter (a) and short diameter (b) of tumors in mice. Tumor volume (mm^3^) was computed as ab^2^/2.24 h after the final administration, mice were anesthetized with an intraperitoneal injection of 1% pentobarbital sodium (40 mg/kg) and then euthanized by cervical dislocation (Percie du Sert et al., 2020). The subcutaneous tumors were then collected, photographed, processed for histopathological sectioning, and utilized for calculating the tumor inhibition rate.

### 2.26 Statistical methods

Statistical analyses were performed using GraphPad Prism software 9.5. Data are presented as the mean ± SD. The normality of the data distribution was verified using the Shapiro-Wilk test. Homogeneity of variances was assessed with Bartlett’s test (or Levene’s test). For comparisons between two groups, an unpaired two-tailed Student’s t-test was used. For comparisons among more than two groups, one-way ANOVA was applied, followed by an appropriate *post hoc* test for multiple comparisons. *P* < 0.05 denoted statistical significance.

## 3 Results

### 3.1 *E. ebracteolata*-containing serum suppresses A549 cells’ proliferation, migration, and invasion capabilities

**FIGURE 2:**
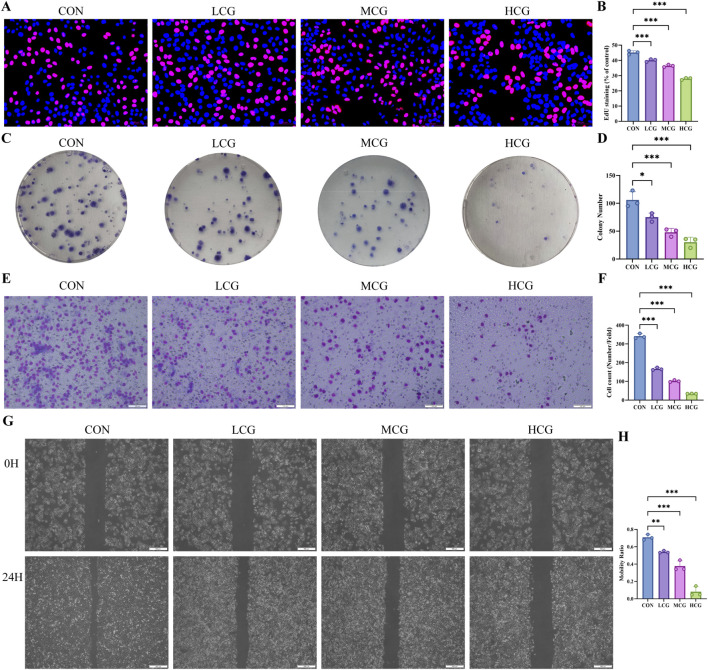
*E. ebracteolata* Medicated Serum Inhibited the Proliferation, Invasion, and Migration of A549 Cells. **(A)** EdU proliferation assay images of A549 cells under Control group (CON), and Low- (LCG), Mid- (MCG), and High-concentration drug-containing serum groups (HCG). **(B)** Quantification of EdU-positive cells from the different treatment groups. **(C)** Colony formation assay images of A549 cells. **(D)** Quantitative results of colony formation assay of A549 cells in different treatment groups. **(E)** Transwell invasion assay images showing invasion cells under different treatments. **(F)** Quantitative results of transwell invasion assay of A549 cells in different treatment groups. **(G)** Wound-healing assay images at 0h and 24 h post-wounding for CON, LCG, MCG, and HCG groups. **(H)** Quantitative results of wound healing assay of A549 cells in different treatment groups. All data, derived from three independent biological replicates, are presented as mean ± SD. One-way ANOVA confirmed the statistical significance. *P < 0.05; **P < 0.01; ***P < 0.001.

Medicated serum was generated in rats and used to incubate A549 cells, with the aim of well elucidating the effects of *E. ebracteolata* on LUAD progression. Proliferation, invasion, and migration were subsequently assessed. According to the EdU assay, the positive rate of A549 cells was lower in the drug-containing serum groups versus the control group, with a gradual decrease in proliferative capacity as the concentration increased (Figures 2A,B). According to the clonogenic assay, serum containing *E. ebracteolata* extract markedly suppressed A549 cells’ capability of forming colonies. The reduction in colony numbers was dependent on concentration (Figures 2C,D). The Transwell assay (Figure 2E) demonstrated a significant inhibition of cell invasive capacity with increasing drug concentrations (Figure 2F). Furthermore, the scratch wound healing assay (Figure 2G) showed a concentration-dependent reduction in A549 cell migration rate (Figure 2H). Taken together, *E. ebracteolata* dose-dependently inhibited the proliferation, migration, and invasion of A549 cells.

### 3.2 Network analysis

**FIGURE 3:**
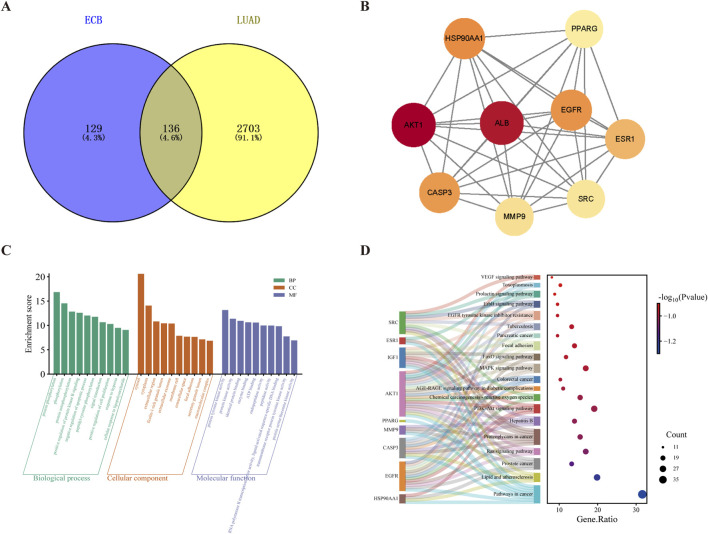
Network analysis and toxicological profiling of ECB against LUAD. **(A)** Venn diagram of ECB and LUAD targets. **(B)** The top 9 core genes were obtained from PPI network analysis. **(C)** GO enrichment analysis and **(D)** KEGG enrichment analysis of ECB-LUAD common targets.

We identified 265 candidate targets of ECB after retrieving the SwissTargetPrediction and PharmMapper databases, and yielded 2,839 disease-related targets for LUAD from the GeneCards and OMIM databases. Intersection analysis revealed 136 shared targets, representing potential therapeutic targets of ECB against lung adenocarcinoma (Figure 3A). The PPI network was constructed by setting the minimum required interaction score to 0.400, corresponding to a medium confidence threshold, to define the edges included in the network. The resulting network comprised 136 nodes and 1,438 edges, with an average node degree of 21.1. The PPI enrichment p-value for the network was less than 1.0e-16, indicating a statistically significant abundance of interactions beyond what is expected by chance, which suggests strong biological relevance among the analyzed proteins. The PPI network constructed based on the STRING database was imported into Cytoscape 3.10.0. The network encompasses 136 nodes and 1,438 interaction edges, and its node size and color intensity were in direct proportion to their degree values (Supplementary Figure 1). We identified nine core targets based on DC, BC, and CC (Figure 3B). The 136 shared targets were subjected to functional enrichment analysis by utilizing the DAVID database. GO analysis confirmed 479 biological process (BP) terms, predominantly associated with protein phosphorylation and autophosphorylation. Cellular component (CC) analysis identified 59 terms, with significant enrichment in the cytosol, cytoplasm, and extracellular region. The 108 molecular function (MF) terms encompassed protein kinase activity, protein tyrosine kinase activity, and identical protein binding (Figure 3C). Through KEGG pathway analysis, 153 enriched pathways were identified, with the top 20 visualized in a bubble plot. Darker blue indicates smaller P-values, and bubble size represents the gene count in the pathway. Key pathways included Pathways in Cancer, Lipid and atherosclerosis, Prostate cancer, Ras signaling pathway, and Proteoglycans in cancer (Figure 3D).

### 3.3 Molecular docking and CETSA experiments

**FIGURE 4:**
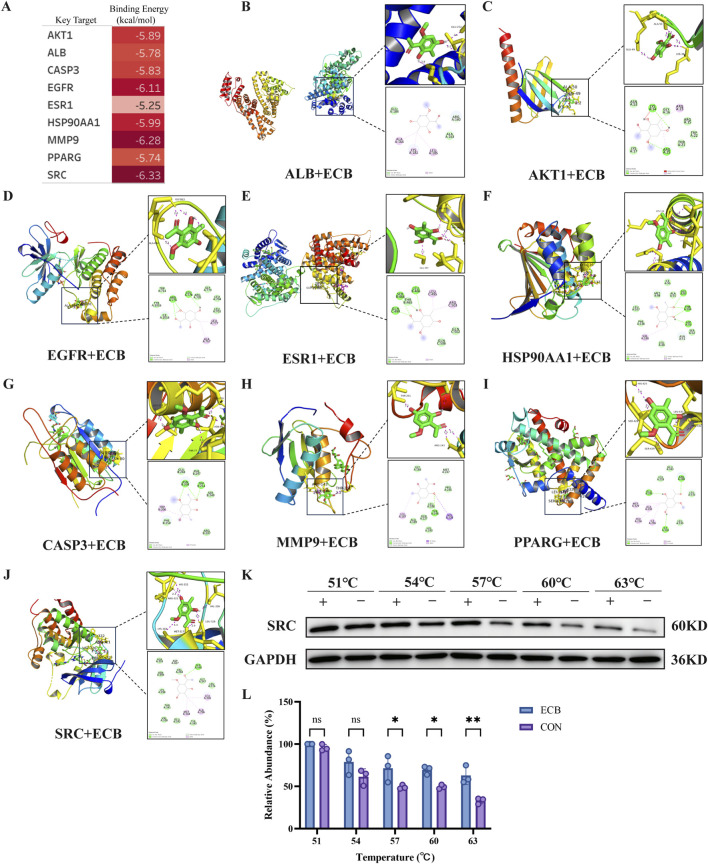
Molecular Docking and CETSA of ECB with LUAD Core Targets. **(A)** Binding energy from molecular docking. Molecular Docking Diagram of **(B)** ALB with ECB; **(C)** AKT1 with ECB; **(D)** EGFR with ECB; **(E)** ESR1 with ECB; **(F)** HSP90AA1 with ECB; **(G)** CASP3 with ECB; **(H)** MMP9 with ECB; **(I)** PPARG with ECB; **(J)** SRC with ECB. **(K)** CETSA investigating the thermal stability of the interaction between ECB and SRC at temperatures ranging from 51 °C to 63 °C. **(L)** Changes in relative protein abundance under different temperatures. All data, derived from three independent biological replicates, are presented as mean ± SD. One-way ANOVA confirmed statistical significance. *P < 0.05; **P < 0.01.

Molecular docking was performed to assess the potential for interaction between ECB and the core targets identified from network analysis (ALB, AKT1, EGFR, ESR1, HSP90AA1, CASP3, MMP9, SRC, PPARG). The binding energies for all targets were below −5.0 kcal/mol (1 kcal ≈4.18585 kJ), which served as an initial screening criterion (Figure 4A). Notably, ECB exhibited the strongest affinity for SRC with a binding energy of −6.33 kcal/mol, which was significantly lower than that of the control compound (−4.29 kcal/mol), suggesting superior binding specificity for SRC. The docking poses were visualized using PyMOL and Discovery Studio (Figures 4B–J).

To validate this prediction and assess binding specificity, CETSA experiments were performed on the core targets identified through network analysis. The CETSA experiments revealed that ECB binding stabilized SRC protein degradation rates across different temperatures (Figure 4K). Starting at 57 °C, the protein abundance in the ECB group became significantly higher than that in the CON group, and this difference was most pronounced at 63 °C (Figure 4L). This provides direct biophysical evidence for a specific interaction between ECB and SRC within cells, validating the docking prediction.

### 3.4 Drug-likeness evaluation and safety assessment

SwissADME database parameters indicated that ECB complies with all criteria of Lipinski’s Rule of Five, suggesting favorable oral bioavailability potential. Toxicity endpoints and overall risk prediction for ECB were conducted using the ProTox-II database. A compound is deemed non-toxic if its toxicity class is ≤3 and the number of positive toxicity endpoints does not exceed three. ECB met these criteria, confirming its safety profile (Supplementary Figure 2).

### 3.5 ECB inhibits LUAD cells’ proliferation, invasion, and migration

**FIGURE 5:**
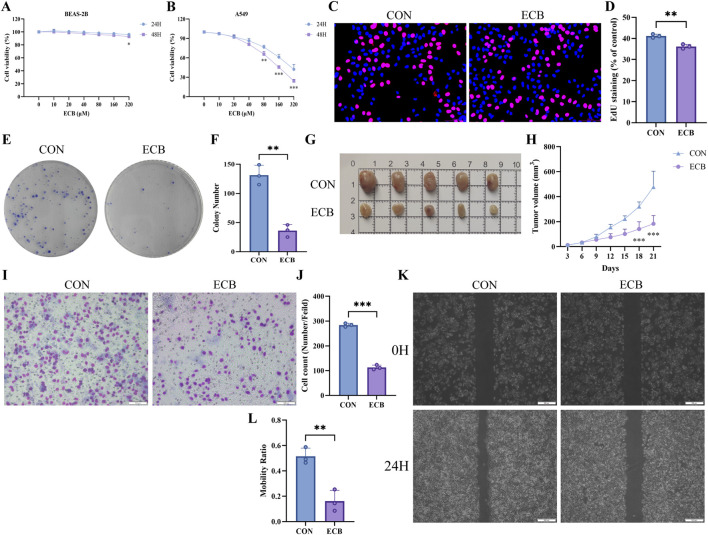
ECB Inhibited the Proliferation, Invasion, and Migration of A549 Cells. **(A)** Effects of ECB on BEAS-2B cells proliferation. **(B)** Viability of the A549 cells treated with varying concentrations of ECB in different time periods. **(C)** EdU proliferation assay images of A549 cells in different treatment groups. **(D)** Quantitative analysis of EdU in A549 cells. **(E)** Representative images of contact dependent on the formation of clones. **(F)** The number of clones formed by A549 cells. **(G)** Representative tumor images in different treatment groups. **(H)** Tumor growth curves across treatment groups. **(I)** Representative images of the Transwell invasion assay using A549 cells. **(J)** Quantitative analysis of cell invasion in Transwell assays. **(K)** Representative result of wound healing assays in A549 cells after 24 h of ECB treatment. **(L)** Quantitative results of wound healing assay of A549 cells in different treatment groups. All data, derived from three independent biological replicates, are presented as mean ± SD. One-way ANOVA confirmed statistical significance. *P < 0.05; **P < 0.01; ***P < 0.001.

With the objective of examining how ECB affected normal human lung epithelial cells, we initially evaluated its impact on BEAS-2B cells’ proliferation and viability by virtue of the CCK-8 assay. According to the analysis results, ECB did not well affect BEAS-2B cells viability at any concentration tested, with the exception of 320 μM after 48 h of treatment (Figure 5A). In contrast, ECB significantly inhibited A549 cell viability starting from 80 μM at 24 h (*P* < 0.01), showing both dose- and time-dependent characteristics. The calculated IC_50_ value of ECB-treated A549 cells at 48 h was 134.2 μM (Figure 5B). Based on these findings, we selected a concentration of 160 μM ECB for subsequent experimental interventions. We performed the EdU proliferation assay and colony formation assay to explain its effect on cell proliferation. The EdU assay revealed remarkably weakened cell proliferation in the ECB-treated group versus the control (Figures 5C,D). In contrast, the colony formation assay showed that ECB treatment greatly reduced the proliferation and tumor-forming abilities of A549 cells (Figures 5E,F). Furthermore, we built a nude mouse xenograft model to further ascertain the *in vivo* inhibitory effect of ECB against the A549 cells proliferation. The ECB-treated group exhibited significantly smaller tumor volumes compared to the control group (Figures 5G,H). According to Transwell invasion assay, the ECB-treated group possessed less invasive cells relative to the control (Figures 5I,J). After 24 h of ECB treatment, the wound healing capacity of A549 cells was notably inhibited versus the control group (Figures 5K,L), suggesting that ECB attenuates A549 cells’ migration. Overall, ECB is effective in reducing A549 cells growth, invasion, and migration.

### 3.6 ECB promotes ferroptosis in A549 cells

**FIGURE 6:**
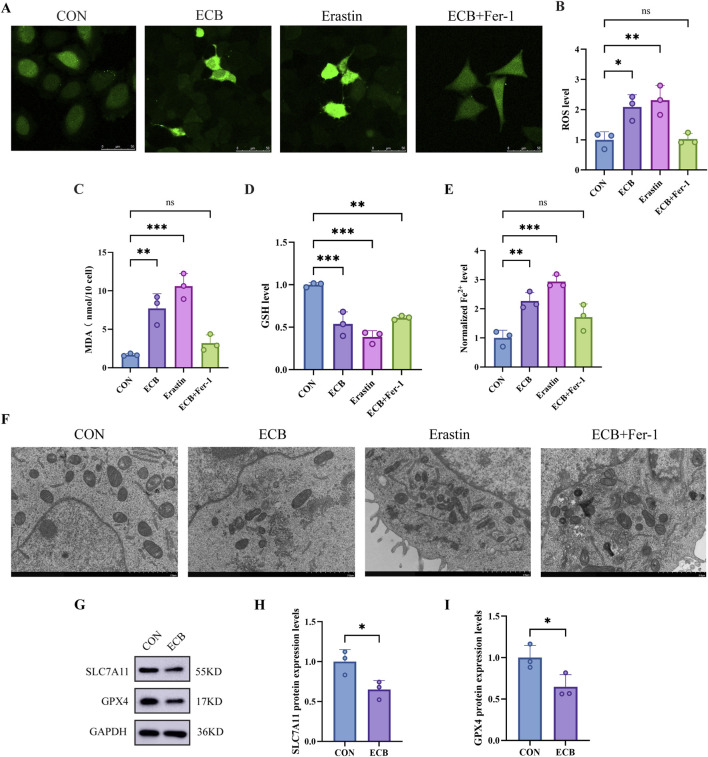
ECB induces ferroptosis in A549 cells. **(A)** Representative confocal microscopy images showing ROS detection in different treatment groups. **(B)** ROS levels in A549 cells. **(C)** Comparison of MDA content in A549 cells among different treatment groups. **(D,E)** Relative MDA and GSH levels in A549 cells. **(F)** Morphological Features of A549 Cells Under TEM. **(G)** Western blot examination of the protein expression of SLC7A11 and GPX4 in A549. **(H,I)** Relative expression level of SLC7A11 and GPX4 protein. All data, derived from three independent biological replicates, are presented as mean ± SD. One-way ANOVA confirmed statistical significance. *P < 0.05; **P < 0.01; ***P < 0.001.

The fluorescent probe DCFH-DA served for assessing intracellular ROS levels in A549 cells. According to Figure 6A, the ECB treatment group triggered obviously higher ROS levels versus the control group, a phenomenon that was also observed in the Erastin group, serving as a positive control. This implies that ECB promotes ROS production. Notably, this ECB-induced ROS increase was significantly suppressed by the co-treatment with the Fer-1 (Figure 6B). Additionally, ECB treatment elevated MDA content and reduced GSH levels in A549 cells, which were also mitigated by Fer-1 co-treatment (Figures 6C,D). Furthermore, Fe^2+^ accumulation was markedly increased in ECB-treated cells, similar to the inducer group, and this accumulation was attenuated by Fer-1 (Figure 6E). Mitochondria in control cells exhibited normal morphology and structure. In contrast, ECB-treated A549 cells and the inducer-treated cells displayed characteristic morphological features indicative of ferroptosis, including mitochondrial shrinkage, increased matrix density, membrane rupture, and reduction in the number of cristae. Importantly, the Fer-1 + ECB group effectively preserved mitochondrial integrity, preventing these ultrastructural changes (Figure 6F). According to Western blot analysis, the ECB-treated group demonstrated lower SLC7A11 and GPX4 expression versus the control group (Figures 6G–I), suggesting that ECB promotes ferroptosis in A549 cells. These results demonstrate that ECB induces Fe^2+^ accumulation, disrupts the antioxidant system, promotes lipid peroxidation, and ultimately triggers ferroptosis in A549 cells.

## 4 Discussion

Serum pharmacology provides experimental conditions that resemble the *in vivo* metabolic and absorptive environment of drugs, thereby reducing interference with experimental outcomes by the physicochemical properties of TCM (Wang et al., 2021). The resulting medicated serum thus more accurately reflects the bioactive metabolites *in vivo*, making it a valid bridge connecting *in vitro* and *in vivo* studies (Gu et al., 2022; Huang W. et al., 2022). Therefore, we first employed drug-containing serum to study the pharmacological effects and mechanisms of *E. ebracteolata in vitro* experiments. As a TCM, *E. ebracteolata* possesses significant medicinal value and demonstrates important research potential and clinical prospects in antitumor applications (Yang et al., 2021; Zhao et al., 2022). Numerous active compounds are isolated from *E. ebracteolata* extracts (Zhao et al., 2022; Yang et al., 2021). For instance, Bisebracteolasins A and B were shown to potently inhibit the proliferation of colorectal cancer cells (CRC) (Yuan et al., 2017). Serum pharmacological analysis demonstrated that A549 cells treated with the drug-containing serum group exhibited remarkably lower proliferation, invasion, and migration rates versus those in the blank serum group, therefore, the *E. ebracteolata*-containing serum markedly inhibited A549 cells’ proliferation, invasion, and migration capabilities.

Subsequently, based on the “disease-gene-target-drug” multi-target and multi-pathway characteristics of network analysis, we identified key targets of ECB in LUAD treatment by virtue of systematic analysis and screening. Molecular docking was employed as a preliminary screening tool to explore putative interactions between ECB and key targets derived from network analysis, including SRC, MMP9, and EGFR. Importantly, the direct interaction between ECB and SRC was experimentally validated by CETSA, which showed that ECB significantly increased the thermal stability of SRC protein, confirming their physical binding. Although network analysis predicted multiple potential targets and pathways, including SRC, these findings served primarily as hypothetical starting points. To move beyond these predictions, we specifically validated the direct binding between ECB and the SRC protein using CETSA experiments. This narrows the focus of the discussion from the broad and potentially questionable “multi-target, multi-pathway” model to SRC, a single, experimentally validated anchor target.

Recent studies have identified more natural compounds with inhibitory effects on tumor progression (Miao et al., 2023; Naeem et al., 2022). ECB, a natural monomer extracted from *E. ebracteolata*, was verified by CCK-8 assays to inhibit the viability of A549 cells while exhibiting non-significant toxicity towards human normal lung epithelial BEAS-2B cells. Utilizing both *in vitro* and *in vivo* experimental models, we demonstrated that ECB effectively suppressed the process of A549 cells being proliferated, invaded and migrated, meanwhile significantly inhibiting the growth of subcutaneous xenograft tumors in nude mice.

As a non-receptor tyrosine kinase, SRC crucially regulates tumor progression (Su et al., 2025). Its overexpression or aberrant activation is closely associated with malignant behaviors such as invasiveness, metastasis, and poor prognosis in lung cancer, BC, and CRC (Kim et al., 2009). Studies indicate that CCL18 promotes proliferation, migration, invasion, chemoresistance, and EMT in low-metastatic BC cells by stimulating high-metastatic BC cells to secrete miR-760-enriched exosomes. The underlying mechanism may involve miR-760 targeting ARF6, thereby triggering the activation of Src/PI3K/Akt signaling pathway (Huang X. et al., 2022). By activating SRC, α6β4 integrin suppresses ferroptosis through a mechanism potentially involving STAT3-mediated transcriptional repression of ACSL4 (Brown et al., 2017). Furthermore, SRC maintains cell survival by regulating the process that lipid metabolism-related genes are expressed (Yang et al., 2022). Additionally, by targeting SRC, Asiatic acid induces ferroptosis in NSCLC cells (Li et al., 2024). Therefore, we propose that ECB inhibits LUAD via ferroptosis.

During ferroptosis, cancer cells exhibit hallmark morphological and biochemical alterations, including mitochondrial shrinkage, diminished mitochondrial cristae, elevated MDA and Fe^2+^ levels, coupled with GSH depletion (Cig et al., 2025; Li et al., 2020). GPX4 (glutathione peroxidase 4) can pivotally regulate ferroptosis (Ursini and Maiorino, 2020) and excels in weakening lipid peroxides to maintain cellular redox homeostasis (Liang et al., 2022). Lipid ROS accumulates when it is depleted or directly inactivated, thereby initiating ferroptosis (Yang et al., 2014). SLC7A11 (Solute Carrier Family 7 Member 11), a cystine/glutamate antiporter, facilitates extracellular cystine uptake to fuel GSH biosynthesis, thereby enhancing cancer cells’ oxidative stress-resistance ability (Yan et al., 2023). In tumor contexts, pharmacological inhibition or genetic suppression of SLC7A11 disrupts cystine flux, precipitating GSH exhaustion and lipid peroxidation cascade, which ultimately activates the ferroptotic death program (Lee and Roh, 2022).

As expected, we observed that ECB treatment significantly triggered ferroptosis in A549 cells, resulting in ROS accumulation, GSH depletion, and elevated levels of MDA and Fe^2+^, a phenotype consistent with that induced by the classical ferroptosis inducer Erastin. TEM also revealed typical morphological features of ferroptosis in A549 cells, including increased mitochondrial membrane density, mitochondrial volume shrinkage, and diminished cristae. More importantly, these ECB-induced morphological and biochemical alterations were largely abrogated by co-treatment with the Fer-1. Additionally, ECB could induce expression downregulation of SLC7A11 and GPX4, as shown by Western blot analysis.

Collectively, this study provides serum pharmacological evidence demonstrating that *E. ebracteolata*-containing serum effectively suppresses the proliferative, invasive, and migratory capacities of A549 cells. Both *in vitro* and *in vivo* experimental validation confirmed ECB’s ability to not only inhibit A549 cells proliferation, invasion, and migration but also potently induce ferroptosis. Our findings provide novel possibilities for ECB in LUAD treatment, well assisting researchers in developing new therapeutic agents and innovative therapeutic strategies for LUAD.

## Data Availability

The datasets presented in this study can be found in online repositories. The names of the repository/repositories and accession number(s) can be found in the article/Supplementary Material.
