# Supplementary figures and images for: Ebracteolata cpd B causes ferroptosis and inhibits progression of lung adenocarcinoma
Source: Front Pharmacol. 2026 Apr 29;17:1706564. doi: 10.3389/fphar.2026.1706564 (PMC13168164; doi:10.3389/fphar.2026.1706564)

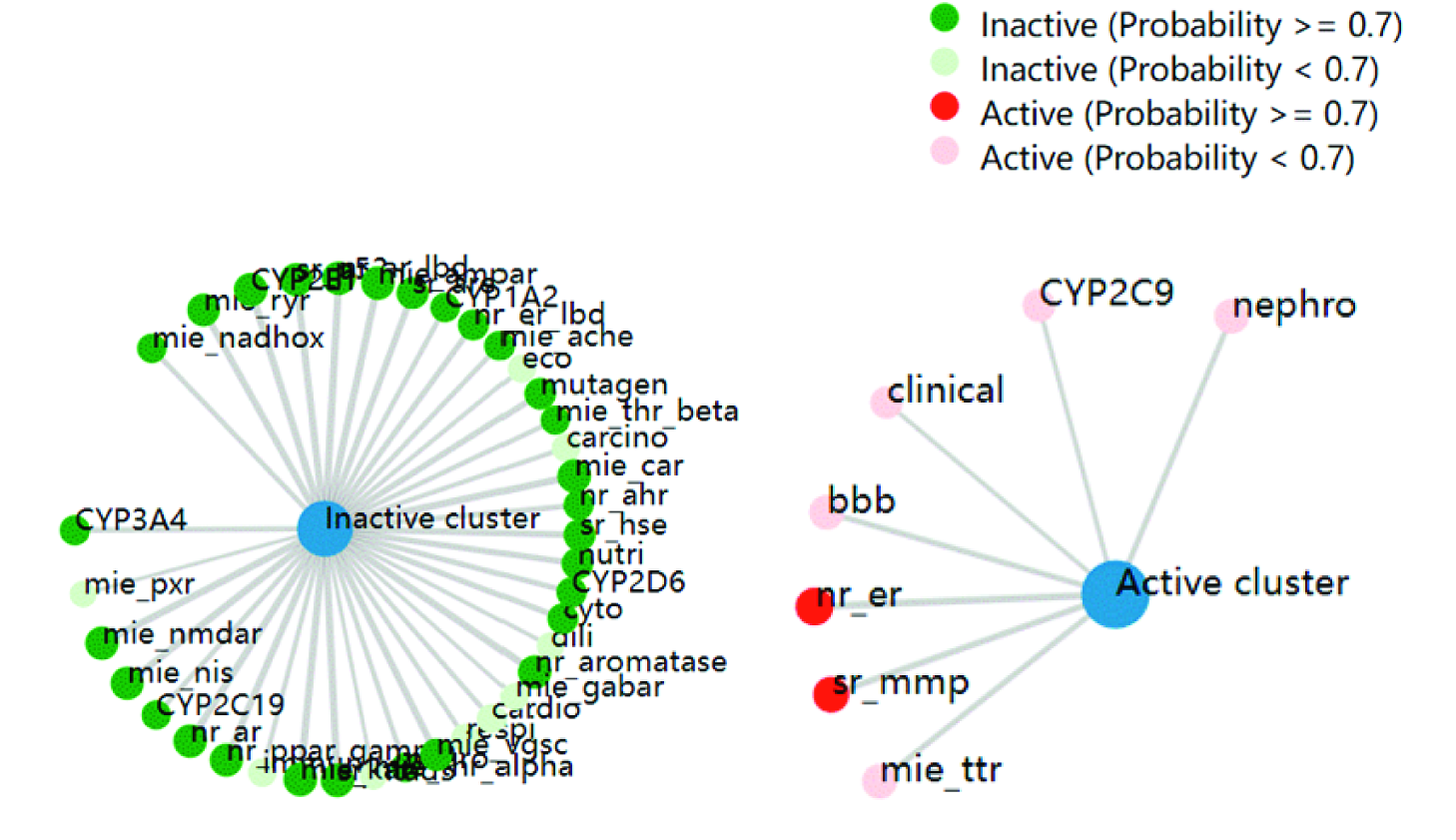

Supplement: Supplementary file 1 [file Image2.tif]

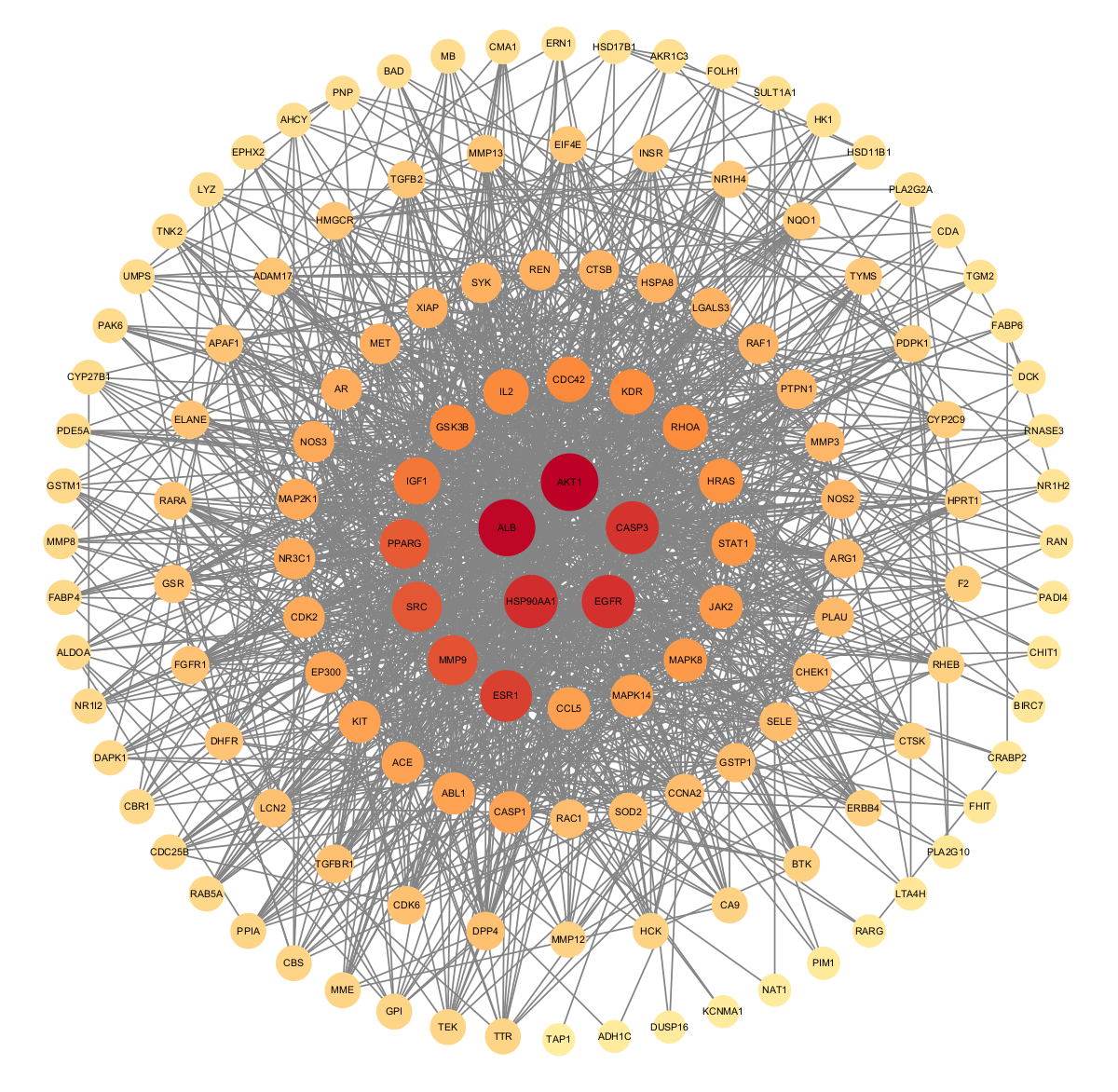

Supplement: Supplementary file 2 [file Image1.png]
